# A planar chiral meta-surface for optical vortex generation and focusing

**DOI:** 10.1038/srep10365

**Published:** 2015-05-19

**Authors:** Xiaoliang Ma, Mingbo Pu, Xiong Li, Cheng Huang, Yanqin Wang, Wenbo Pan, Bo Zhao, Jianhua Cui, Changtao Wang, ZeYu Zhao, Xiangang Luo

**Affiliations:** 1 State Key Laboratory of Optical Technologies on Nano-Fabrication and Micro-Engineering, Institute of Optics and Electronics, Chinese Academy of Science, P. O. Box 350, Chengdu 610209, China

## Abstract

Data capacity is rapidly reaching its limit in modern optical communications. Optical vortex has been explored to enhance the data capacity for its extra degree of freedom of angular momentum. In traditional means, optical vortices are generated using space light modulators or spiral phase plates, which would sharply decrease the integration of optical communication systems. Here we experimentally demonstrate a planar chiral antenna array to produce optical vortex from a circularly polarized light. Furthermore, the antenna array has the ability to focus the incident light into point, which greatly increases the power intensity of the generated optical vortex. This chiral antenna array may have potential application in highly integrated optical communication systems.

Optical vortex, referring to the light beam carrying orbital angular momentum (OAM), has attracted great interest for its helical phase front comprising an azimuthal phase term exp(*ilΦ*)^1^, where *l* is the topological charge, and *Φ* is the azimuthal angle. Dissimilar to the spin angular momentum (SAM), which has only two values of ±*ħ* (Plank’s constant divided by 2π) per photon, the topological charge *l* provides theoretically unlimited states for OAM. Therefore, it is important for optical communication as the spiral phase front can carry additional information^2^,^3^,^4^,^5^,^6^,^7^, which provides tremendously extra data capacitance and can effectively release the rapid achieving limitation of optical communication. In recent years, varies of studies on optical vortex have been reported and applications based on optical vortex have been introduced in the areas of physics detection, optical manipulation, and biology microscopy for its obvious advantages^8^,^9^,^10^,^11^.

Typically, the helical phase distributions of optical vortices are generated by spiral phase plates (SPPs)^12^, computer induced holograms^13^, subwavelength gratings^14^, annular gratings^15^, and nonlinear medium like crystal liquid^16^,^17^,^18^,^19^,^20^,^21^. Appearing in last decades, metamaterials have been explosively developed in manipulating electromagnetic wave, and have provided new tubes to achieve characters non-exist in natural occurred materials^22^,^23^,^24^,^25^,^26^,^27^,^28^. As a particular property of metamaterial, phase discontinuity is recently proposed to achieve anomalous reflection and transmission of light. In fact, a series of flat optical devices have been elaborately designed several years ago to modulate the Snell’s law and realize functionalities such as abnormal beam deflection, subwavelength focusing and imaging^29^,^30^. These plasmonic devices are the early version of meta-surfaces. Subsequently, various metamaterials, “V” shaped antennas for example, have been introduced to induce cross components and phase shift by tuning the shape and orientation of each antenna for linearly polarized incidence^31^,^32^,^33^. For circularly polarized light, it becomes much easier: a dipole antenna was demonstrated to be able to manifest such effect near perfectly^34^. Unfortunately, all the above two kinds of antennas are suffering the low efficiency due to the intrinsic limitation of single layer meta-surface. At best 25% of the total energy can be utilized, which is not anticipated in many applications. Due to the low conversion efficiency of these applications, co-polarized component still exists in the outgoing field. And cascaded quarter and half wave plates are typically required in order to filter the co-polarized components. This may increase the complexity of the system and introduce extra loss.

In this paper, we demonstrate a chiral meta-surface to convert a circularly polarized light into an optical vortex and focus the converted light to a predicated point. The meta-surface tunes the phase distribution of the incident beam based on the concept of discontinues phase modulation. Consequently, the proposed meta-surface has two main functions. Firstly, with a circularly polarized incident beam, the meta-surface converts it into a beam carrying OAM. Secondly, the incident field can be focused into a point with a certain focus length. The total energy coefficient of this meta-surface exceeds 40%. By properly designing the phase modulation distribution, the co-polarized component of the incident field does not participate in the focusing process. Therefore, only optical vortex can be observed in the focused field. The thickness of this planar chiral meta-surface is less than λ/4, thus has potential application in compact integrated optical systems.

## Results

### Simulated results

**Figure 1:**
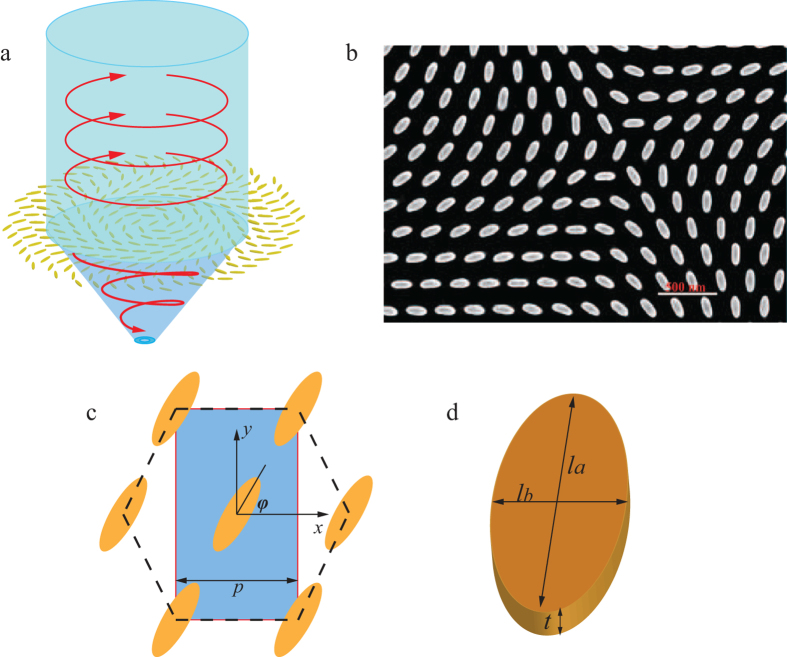
Structural geometry and SEM image of the chiral meta-surface. (**a**) Schematic view of the meta-surface for OAM converting and focusing. The incident planar circularly polarized field is converted into a focused light carrying OAM. (**b**) The scanning electron microcopy image of the fabricated sample which converts the incident CP light into a focused light carrying OAM with topological charge *l *= 2. (**c**) Unit cells of the meta-surface. The array elements are hexagonally arranged. Period in *x*-direction is *p *= 280 nm. (**d**) The long axis and short axis of the elliptical cylinder antenna are respectively *l*_*a*_* *= 220 nm and *l*_*b*_* *= 66 nm. The thickness of the antenna is *t *= 180 nm.

The polarization conversion and focusing process of the meta-surface is schematically shown in Fig. 1a. The details of the meta-surface are depicted in Fig. 1c and 1d. It is composed of elliptical nano-antennas oriented along certain directions. The elements are hexagonally placed, and the period along *x* direction is defined as *p *= 280 nm and thus the period in *y* direction is 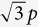. The length of the long axis and short axis of each elliptical antenna are respectively *l*_*a*_* *= 220 nm and *l*_*b*_* *= 66 nm. Each element is oriented along its normal axis with an angle *ϕ* with respect to +*x* direction. All of the geometry parameters are optimized using the commercial electromagnetic simulation software of CST Microwave Studio 2014. One can see that the meta-surface is anisotropic, and has the function of partially converting the incident circularly polarized light into cross-polarized one.

**Figure 2:**
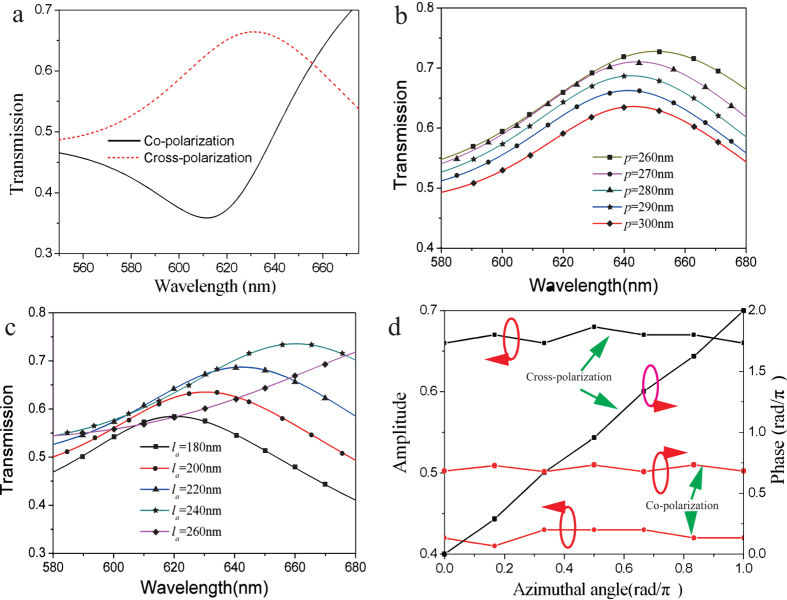
Transmitted amplitude and phase property of the meta-surface. (**a**) Transmission spectra of co-polarization and cross-polarization under the condition of circularly polarized incidence. (**b**) Transmission character of cross-polarization versus the period *p* of the antenna array. (**c**) Transmission spectra of cross-polarization versus *l*_*a*_ while keeping *l*_*b*_/*l*_*a*_* *= 0.3. (**d**) The transmitted amplitude and phase of co-polarization and cross-polarization versus the orientation angle at the wavelength of 633 nm.

We firstly simulated and optimized the transmission character of the meta-surface. The permittivity of silver and dielectric are taken from experimental results^35^. With a right-handed circularly polarized (RHCP) incidence, the co-polarized and cross-polarized transmissions are calculated, as shown in Fig. 2a. We observe the discrepancy in the transmission spectra, elucidating the anisotropic property of the optical meta-surface. Notable, in the range 500–655.8 nm, the transmission of cross-polarization is higher than that of co-polarization component, and the cross polarization transmission reaches its maximum at 633 nm. It demonstrates that the incident RHCP light is almost converted into left-handed circularly polarized (LHCP) light ignoring the reflection and absorption caused by the lossy metal. This is in contrast to the meta-surfaces based on plasmonic V-shaped antenna, where the majority of scattered light remains in the ordinary component (co-polarized), resulting in low signal-to-noise ratio (SNR)^31^. Parameters of the nano-antenna are optimized to obtain good cross-polarization transmission character. The period and the size of the nano-antenna are varied in the simulation, as depicted in Fig. 2b and 2c. When the period *p* gets larger, the resonant frequency has blue shift and the cross-polarization transmission decreases at 633 nm. However, the transmission of the LHCP light is fluctuant when the size of the antenna changes. We increase *l*_*a*_ from 180 nm to 260 nm by the step of 20 nm while keeping the ratio *l*_*b*_/*l*_*a*_ as a constant of 0.3. The transmission of LHCP light reaches its peak at 633 nm when *l*_*a*_ equals to 220 nm. Therefore, as a tradeoff, the values of *p* and *l*_*a*_ are set as 280 nm and 220 nm, respectively.

As shown in Fig. 2d, the phase of the cross-polarization component, namely LHCP, is linear corresponding to the orientation angle **ϕ**, and the gradient is 2 according to the simulated results. Thus, the transmitted phase of cross-polarization varies in the range from 0–2π when the optical antenna rotates along its normal axis with the orientation angle *ϕ* increase from 0 to π. It is worthy to note that the transmitted phase of the co-polarized component, i.e. RHCP, is almost a constant insensitive to the orientation angle **ϕ**, as shown in Fig. 2d. Subsequently, we utilize this phase controllable meta-surface to construct a focused OAM light generator by properly arranging each antenna element with a rotation angle around its cylinder axis.

Theoretically, in the sake of converting the incident planar circularly polarized light into a focused light carrying OAM, two basic requirement need to be satisfied for the phase distribution of the meta-surface. One is the wave vector along azimuthal direction to produce OAM, and it can be easily observed in circularly polarized waves. The other one is the parabola phase distribution along radial direction. Normally, the phase distribution of the meta-surface could be stated in polar coordinate as:


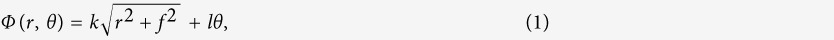


where *κ* is the wave vector, *r* is the distance from the coordinate origin, and *f* is the focus length of the meta-surface. Parameter *l* represents the topological charge of the radiated OAM, and *θ* is the azimuthal angle. It can be concluded that the phase distribution contains two main function components. One is the helical phase front 
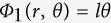
 to produce the OAM light. The other one is the phase distribution component 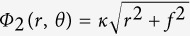 for focusing the converted OAM light to a point with focus length *f*. It is also notable that if we define the value of Ф as a constant, then the phase front isoline can be described as follows,


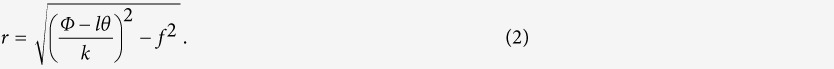


Specially, when *f* is infinite, the formula (2) can be simply written as *r *= *lθ/k*, which is the classical Archimedean spiral.

Based on the aforementioned analysis, two meta-surfaces with radius of 20 μm for OAM focusing are designed and simulated using full-wave electromagnetic simulation. In the characteristic simulation, the focus length *f* is designed to be 10 μm and the topological charges are respectively 1 and 2. Each antenna element rotates along its normal axis with an angle of Ф/2 according to formula (1). A planar RHCP light is employed as the incident source. It can be predicted that most part of the incident RHCP light is transformed into light carrying OAM and focused at the point with 10 μm to the meta-surface in the normal axis. However, as the phase distribution of the transmitted co-polarization component is insensitive to the meta-surface, the propagation direction of the LHCP component in the outgoing field does not change and will depart off the focus. Therefore, optical vortex is the main constituent involved in the zone around the focus.

**Figure 3:**
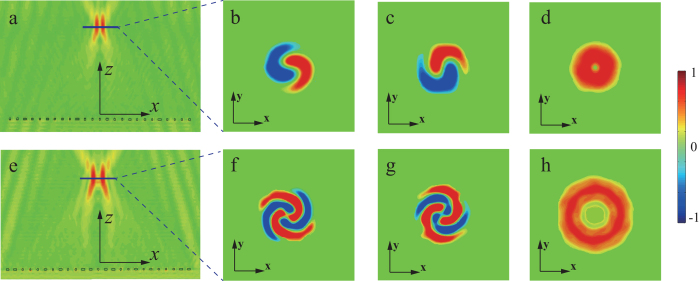
Simulated electric field distribution of the generated optical vortex beam for *l *= 1, 2. (**a**) The energy intensity of outgoing electric field of converted OAM light with *l *= 1 in *xoz* plane. (**b**)*x*- and (**c**) *y*-component of the instant radiated electric field in *xy* plane at the focus. (**d**) The energy intensity of the radiating electric field for optical vortex with topological charge *l *= 1 in *xy* plane. (**e**) The energy intensity of outgoing electric field in *xoz* plane of the OAM beam with topological charge *l *= 2. (**f**)–(**h**) *x*-, *y*- components and the energy intensity of the generated focused optical vortex with topological charge of *l *= 2 in *xy* plane at the focus, respectively.

The radiated electric field distributions are simulated to elucidate the OAM conversion and focusing character. The cross-section view of the radiated field in *xoz* plane, shown in Fig. 3a, is first simulated to demonstrate the focusing effect of the meta-surface. It clearly shows that the output electric field concentrates into a compact zone around *z*-axis. Subsequently, the *x*- and *y*-component of the instant electric field in *xoz* plane for topological charge *l *= 1 are depicted in Fig. 3b and 3c, respectively. Spiral patterns, indicating the helical phase distribution of the OAM light, can be clearly observed. An annular ring with dark center is obtained for the energy intensity as illustrated in Fig. 3d, which is the direct consequence of optical vortex beam. Furthermore, another meta-surface to convert the incident circularly polarized light into OAM light with *l *= 2 is also simulated, and the electric field distributions are shown in Fig. 3e-3h, which validates the flexibility and the advantage of this chiral meta-surface in manipulating light.

### Experimental results

In experiment, based on the above analysis, a sample of the chiral meta-surface was fabricated to convert the incident circularly polarized light into a focused OAM light with *l *= 2. We first deposited a 180-nm-thin film of silver using magnetron sputter onto a quartz substrate coated with 2-nm of Cr. Then the elliptical optical antennae are created using Focused Ion Beam (FIB, FEI Helios Nanolab 650) lithography and reactive-ion etching. The SEM photo of the sample is depicted in Fig. 1b. The radius of the effective area of the fabricated sample is 20 μm and the focus length *f* is designed as 20 μm for easier measurement.

**Figure 4:**
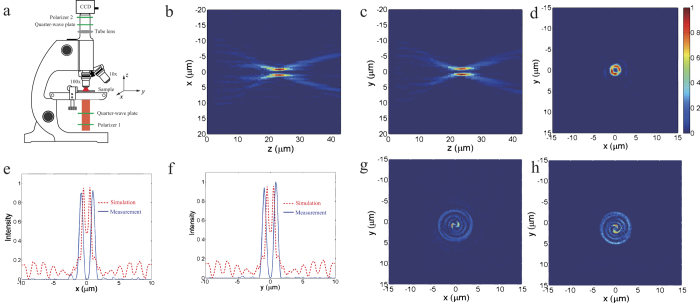
Experiment of the polarization conversion and focusing effect of the fabricated chiral meta-surface. (**a**) Schematic diagram of the experiment setup. Measured power intensity profile of the focused field in (**b**) *xoz* plane, (**c**) *yoz* plane, and (**d**) *xy* plane at z* *= 23 μm. (**e**)-(**f**) The simulated (dashed lines) and measured (solid lines) intensity spectra of the focused field respectively along *x*- and *y*-axis for figure (**d**). (**g**)-(**h**) The *x*- and *y*- component of the focused OAM light.

Subsequently, the radiated electric field intensity was measured using our home-built microscope, as shown in Fig. 4a. The measured intensity patterns of the transmitted light are shown in Fig. 4b-4d. The electric field distributions in *xoz* and *yoz* planes unambiguously validate the focusing ability of the meta-surface, and one can see that the measured focus length is about 23 μm, which is close to the designed value of 20 μm. The energy distribution in the focus plane depicted in Fig. 4d shows that a bright annular ring with minimum power at the center is obtained, further confirming that an OAM light is focused. And the intensity spectra along *x*- and *y*- axis demonstrate that the radius of the annular ring is less than 1.8 μm, as shown in Fig. 4e and 4f. Consequently, the incident energy can be enormously concentrated, which is desired in most optical applications. The red dashed lines in Figs. 4e and 4f represent the simulated energy spectra along *x*- and *y*-direction in the focus plane. It is notable that the power intensity is nonzero in the middle of the spectra, which is due to the co-polarization component is not filtered in the output light. Figure 4g and 4h depict the *x*- and *y*- component of the focused OAM light in the focus plane, in which we can see a pair of spiral, indicating the helical phase distribution of the OAM light.

## Discussion

Typically, this meta-surface can be seen as a composite of a positive lens and a polarization converter. Therefore, it also has the ability to convert a point-radiated incident circularly polarized light into a beamed OAM light with opposite rotation direction according to the optical reciprocal theory. We again simulated this function of this chiral meta-surface. A point source radiating RHCP light is put at the focus of the meta-surface in Fig. 3e. It is predicted that majority of the incident RHCP light is transformed into light carrying OAM of *l *= 2 and the spherical wave is converted into plane wave. However, the propagation direction of the unconverted RHCP component in the outgoing field does not change and will be scattered away from the normal axis.

**Figure 5:**
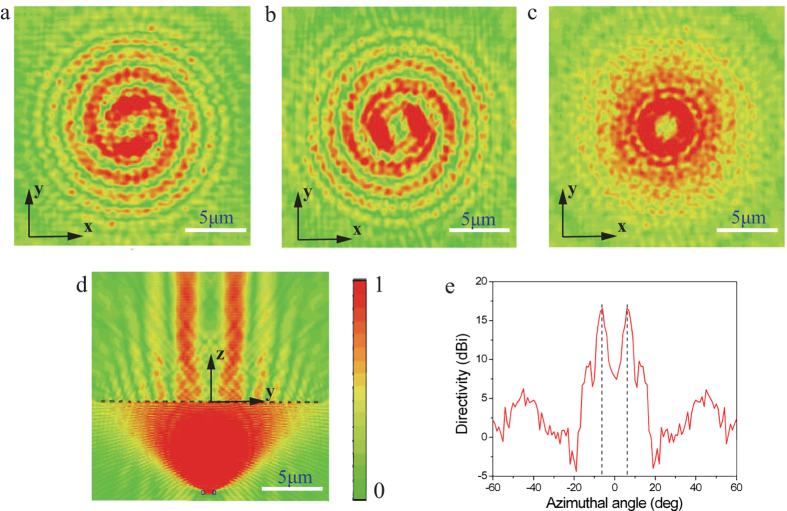
Simulated results of beaming function for the chiral meta-surface. (**a**) *x*- and (**b**) *y*-component and (**c**) power intensity of the transmitted electric field in transverse plane (*xy* plane). (**d**) The energy intensity of the outgoing electric field in *yoz* plane. (**e**) Far-field radiation pattern in *yz* plane.

The simulated electric field distributions for the beaming process are depicted in Fig. 5. Spiral patterns of the electric field for *x*- (Fig. 5a) and *y*-component (Fig. 5b) and annular ring with minimum center for the power intensity (Fig. 5c) are obtained, confirming the production of OAM with *l *= 2. Furthermore, the cross-section view of the radiated field in Fig. 5d demonstrates the beaming effect of the meta-surface. The far-field radiation pattern of the outgoing light shown in Fig. 5e elucidates that the diffusion angle is 6° for this beamed light.

This multi-function meta-surface may be utilized in optical communication systems and is suitable for optical manipulation and trapping. And the concept of this phase discontinued meta-surface can be utilized in enormous applications in wide frequency range by properly scaling the size of the nano-antenna elements.

## Methods

### Fabrication

The fabrication began with depositing a 2-nm-thin film of Cr onto a clean and planar quartz substrate to improve the adhesion between the silver film and the substrate. Then a 180-nm-thin silver film was deposited on the quartz substrate by magnetron sputtering. Subsequently, the elliptical optical antennae are created using Focused Ion Beam lithography in the silver film. The size of the sample is 2 × 2 inch^2^ and the radius of the pattern area is 10 um.

### Measurement

The measurements were carried out using our home-built microscope. First, the incident collimated beam from a HeNe laser at λ* *= 633 nm is converted into RHCP light by a cascaded polarizer and a quarter-wave plate before illuminating onto the sample. The transmitted light passes through a 100 × objective and a 0.65 × tube lens, and then converted into linearly polarization by another quarter-wave plate and filtered by polarizer with polarization direction orthogonal to the first polarizer aiming to filter the co-polarized component. Subsequently, the intensity patterns of the filtered light are imaged by a CCD camera. We set the output surface of the sample as the z* *= 0 plane, and the center of the hollow ring of OAM light in the focused plane as the original for x and y coordinate. Images of the electric field in *xy* plane are taken by moving the objective along z direction with a step of 1 μm. And then the intensity at *y *= 0 in each *xy* plane is extracted and forms the *xz* profile shown in Fig. 4(b).

## Author Contributions

X.L.M, M.B.P and X.L contributed equally to the numerical simulation and physical interpretation. X.L., H.C., W.B.P, Y.Q.W., B.Z., J.H.C., C.T.W. and Z.Y.Z. fabricated the sample and carried out the experiment, X.L.M. and M.B.P. wrote the manuscript. X.G.L. conceived the original idea and supervised the project. All the authors have analyzed and discussed the results thoroughly and contributed to the writing of the manuscript.

## Additional Information

**How to cite this article**: Ma, X. *et al.* A planar chiral meta-surface for optical vortex generation and focusing. *Sci. Rep.* **5**, 10365; doi: 10.1038/srep10365 (2015).
